# Efficacy and safety of hybrid closed-loop systems in children aged < 7 years with type 1 diabetes: a systematic review and meta-analysis of randomized controlled trials

**DOI:** 10.48130/pedi-0026-0006

**Published:** 2026-08-12

**Authors:** Tingting Zhang, Na Wang, Bo Wang, Menghao Wang, Jia Zhang

**Affiliations:** Department of Pediatric, Suqian First Hospital, Suqian, Jiangsu 223800, China

**Keywords:** Child, Hybrid closed-loop, Artificial pancreas, Type 1 diabetes

## Abstract

This study aimed to evaluate the clinical efficacy and safety of hybrid closed-loop systems for glycemic control in children aged < 7 years with Type 1 diabetes. Relevant studies published up to July 30, 2025 were electronically retrieved from the PubMed, Cochrane Library, Embase, and Web of Science databases. Two investigators independently screened the literature, extracted valid data, and assessed the risk of bias of included studies, with a meta-analysis conducted via RevMan 5.4 software. Three randomized controlled trials, with 274 children enrolled, were incorporated into the analysis. The results indicated that 3 months of using the hybrid closed-loop system significantly increased the percentage of time in range (mean difference [MD] = 9.95%, 95% confidence interval [CI] [7.8, 12.09], *p* < 0.00001) and reduced glycated hemoglobin (MD = −0.51%, 95% CI [−0.64, −0.37], *p* < 0.00001). However, no significant difference was noted in the percentage of time below range between the two groups (MD = 0.56%, 95% CI [−0.13, 1.25], *p* = 0.11). A comparison of serious adverse events showed no statistically significant differences in the risk of diabetic ketoacidosis (*p* = 0.50) and severe hypoglycemia (*p* = 0.64) between the hybrid closed-loop system group and the conventional therapy group. The hybrid closed-loop system can effectively improve glycemic control in children under 7 years old with Type 1 diabetes without increasing the risk of adverse events. Further high-quality, large-sample clinical studies are still required to verify and supplement the present conclusions.

## Introduction

Worldwide, there has been a substantial increase in the incidence of Type 1 diabetes (T1D). According to data from the 11^th^ edition of the *Diabetes Atlas*^[1]^, in 2024, the global prevalence count of individuals affected by T1D reached 9.5 million (a 13% increase compared with 2021). Specifically, 1 million of those affected were children aged between 0 and 14 years. The incidence of T1D in children continues to rise, with an annual growth rate of 3%–4%. This trend is particularly pronounced among children under 5 years old, who represent the fastest-growing group of new cases^[2]^. This shift toward an increasingly younger age of onset makes glycemic management in young children more challenging^[3,4]^.

Children aged < 7 years with T1D present distinct clinical characteristics compared with older children and adults: they have lower insulin requirements, higher and more variable insulin sensitivity, unpredictable eating and activity patterns, and nonawareness of hypoglycemia^[5]^. As a result, this population achieves target glycemic control less frequently and is at higher risk of hypoglycemia and diabetic ketoacidosis^[6]^. Although conventional therapies such as sensor-augmented pump (SAP) therapy can partially improve glycemic control^[7]^, achieving the target glucose levels remains difficult in young children^[8,9]^, and they remain at an elevated risk of microvascular complications^[10,11]^. The hybrid closed-loop system (HCL) integrates continuous glucose monitoring (CGM) data with insulin delivery in real time via a control algorithm, enabling more precise and dynamic glycemic regulation^[12]^. Its efficacy and safety have been well established in adolescents and adults^[13−15]^. Studies have shown that the MiniMed 670G system reduced glycated hemoglobin (HbA1c) to 7.1% and increased time in range (TIR) to 73% in children and adolescents^[16]^. A multicenter study on the OmniPod system also demonstrated that children over 6 years old achieved a TIR of 70.1%^[17]^. However, as most commercially available HCL devices are approved for use in individuals aged > 7 years, evidence regarding their use in younger children remains limited. Only one meta-analysis included participants aged 2–7 years, but the sample size was small, and no comprehensive systematic analysis was conducted on outcomes such as HbA1c, TIR, time below range (TBR), or time above range (TAR) in this age group^[18]^.

Given the rapidly increasing global incidence of T1D in young children and the challenges in glycemic management within this population, coupled with the current insufficient evidence regarding HCL therapy in this age group, this meta-analysis aims to fill a critical evidence gap. It focuses specifically on evaluating the glycemic efficacy and safety of HCL in children aged < 7 years, with the objective of supporting clinical decision-making and ultimately enhancing glycemic outcomes in young children.

## Materials and methods

This study was performed in line with a pre-registered protocol [PROSPERO (International Prospective Register of Systematic Reviews): CRD420251137957] and followed the PRISMA (Preferred Reporting Items for Systematic Reviews and Meta-Analyses) guidelines^[19]^. The completed PRISMA 2020 checklist is provided in Supplementary Table S1.

### Search strategy

Two investigators (Tingting Zhang and Na Wang) searched the PubMed, Cochrane Library, Embase, and Web of Science databases from their inception to July 30, 2025. We combined both controlled vocabulary terms and free-text keywords in our search strategy. The key concepts encompassed "child", "hybrid closed-loop or HCL", "artificial pancreas" and "Type 1 diabetes or T1D", along with their synonyms and abbreviations. The search queries were constructed using Boolean logic operators to link the conceptual blocks.The search strategies are provided in Supplemental File S1.

### Inclusion and exclusion criteria

The inclusion criteria for this study were defined as follows: (a) Population: children under 7 years old with a confirmed diagnosis of T1D. The diagnostic criteria for T1D were those defined and reported in each original randomized controlled trial, and no uniform diagnostic standard was newly set in this meta-analysis; (b) Intervention: the intervention group used HCL for 24-h insulin delivery; (c) Comparison: any non-closed-loop insulin therapy regimen; (d) Outcomes: TIR, HbA1c, TBR, TAR, and severe adverse events (SAEs); (e) Study design: limited to randomized controlled trials and a study duration of no less than 12 weeks; (f) Published in English. This meta-analysis restricted inclusion to English-language studies to ensure the accuracy of data extraction, minimize potential bias arising from translation errors, and focus on well-established high-quality evidence in pediatric T1D.

Exclusion criteria were conference abstracts, letters, case reports, reviews, animal experiments, clinical guidelines, and studies with the same participants but different purposes.

### Study selection and data extraction

Tingting Zhang and Na Wang independently screened the titles and abstracts to exclude irrelevant studies. The full texts of the remaining articles were then assessed to ensure that all relevant studies were thoroughly evaluated. Any disagreements between the reviewers would be referred to an independent third author (Bo Wang) for resolution. Data from the included studies was extracted by Tingting Zhang and Bo Wang, and the extracted information was as follows: First author, year of publication, study design, number of patients, duration of diabetes, prior therapy, type of HCL, control group treatment, study duration, and glycemic outcomes.

### Outcome definition

The primary outcome was the TIR, namely the percentage of time spent in the target sensor glucose range (70–180 mg/dL).

The secondary outcomes are specified below: (a) Glycated hemoglobin; (b) Percentage of hypoglycemic time (sensor glucose < 54 mg/dL and < 70 mg/dL); (c) Percentage of hyperglycemic time (sensor glucose > 180 mg/dL, > 250 mg/dL, and > 300 mg/dL).

Additionally, data on safety outcomes were gathered, which included: (a) Severe hypoglycemia (defined as requiring treatment for altered mental status); (b) Diabetic ketoacidosis (DKA).

### Quality assessment of the included studies

Methodological quality was appraised independently by two investigators (Na Wang and Bo Wang) using the Cochrane RoB 2 tool. The assessment covered five core domains defined by the Cochrane guidelines: (a) Adequacy of randomization; (b) Adherence to the intervention protocols; (c) Completeness of outcome data; (d) Objective outcome measurement; (e) Selective reporting avoidance; (f) Overall bias.

Each domain was categorized as low, with a moderate risk or high risk of bias.

### Certainty of evidence assessment

The certainty of the evidence was quantified using the Grading of Recommendations Assessment, Development and Evaluation (GRADE) scale^[20]^.

### Data synthesis and analysis

Statistical analyses were conducted utilizing Review Manager (RevMan) software version 5.4 (The Cochrane Collaboration, Copenhagen, Denmark)^[21]^. For continuous outcome measures, the mean difference (MD) was used to pool the effect sizes; if the variables were not normally distributed, the median Quantiles 1 and 3 (Q1 and Q3) was used for description. For dichotomous outcome measures, the odds ratio (OR) was used as the effect size for data pooling, with the 95% confidence interval (CI) to reflect the sampling error of the effect sizes. Two-tailed tests were used for all analyses, with statistical significance defined as a *p*-value < 0.05.

For data missing from the included studies, we first attempted to contact the original authors via email to obtain supplementary data. If complete data could not be obtained, only studies with available data were included in the meta-analysis, and no imputation for missing data was performed.

Heterogeneity among included studies was evaluated using the χ^2^ test and the I^2^ statistic. Heterogeneity was graded as follows: low heterogeneity for I^2 ^< 25%, moderate heterogeneity for 25% ≤ I^2^ < 50%, and high heterogeneity for I^2^ ≥ 50%. The statistical significance of heterogeneity was defined as *p* < 0.10. All outcomes in this study were analyzed using the random effects model regardless of their heterogeneity level. Sensitivity analyses conducted by sequentially omitting one study at a time were performed to explore potential sources of heterogeneity and to test the robustness of the pooled results.

To verify the stability and reliability of the meta-analysis results, sensitivity analyses were performed for all outcome measures. One study at a time was sequentially excluded, and the remaining studies were re-analyzed using the random effects model. Changes in the pooled effect size, 95% CI, *Z*-value, *p*-value, and heterogeneity (I^2^) were evaluated before and after each exclusion. The result was considered to be stable and reliable if the pooled effect size and statistical significance did not change substantially after the exclusion of any single study. If substantial changes or reversed statistical significance were observed, the source of heterogeneity was explored further.

## Results

### Study selection

In total, 3,951 articles were obtained through preliminary screening. According to the inclusion and exclusion criteria, three studies involving 274 participants were finally selected for inclusion^[22−24]^. The process of their identifcation and selection is shown in Fig. 1.

**Figure 1:**
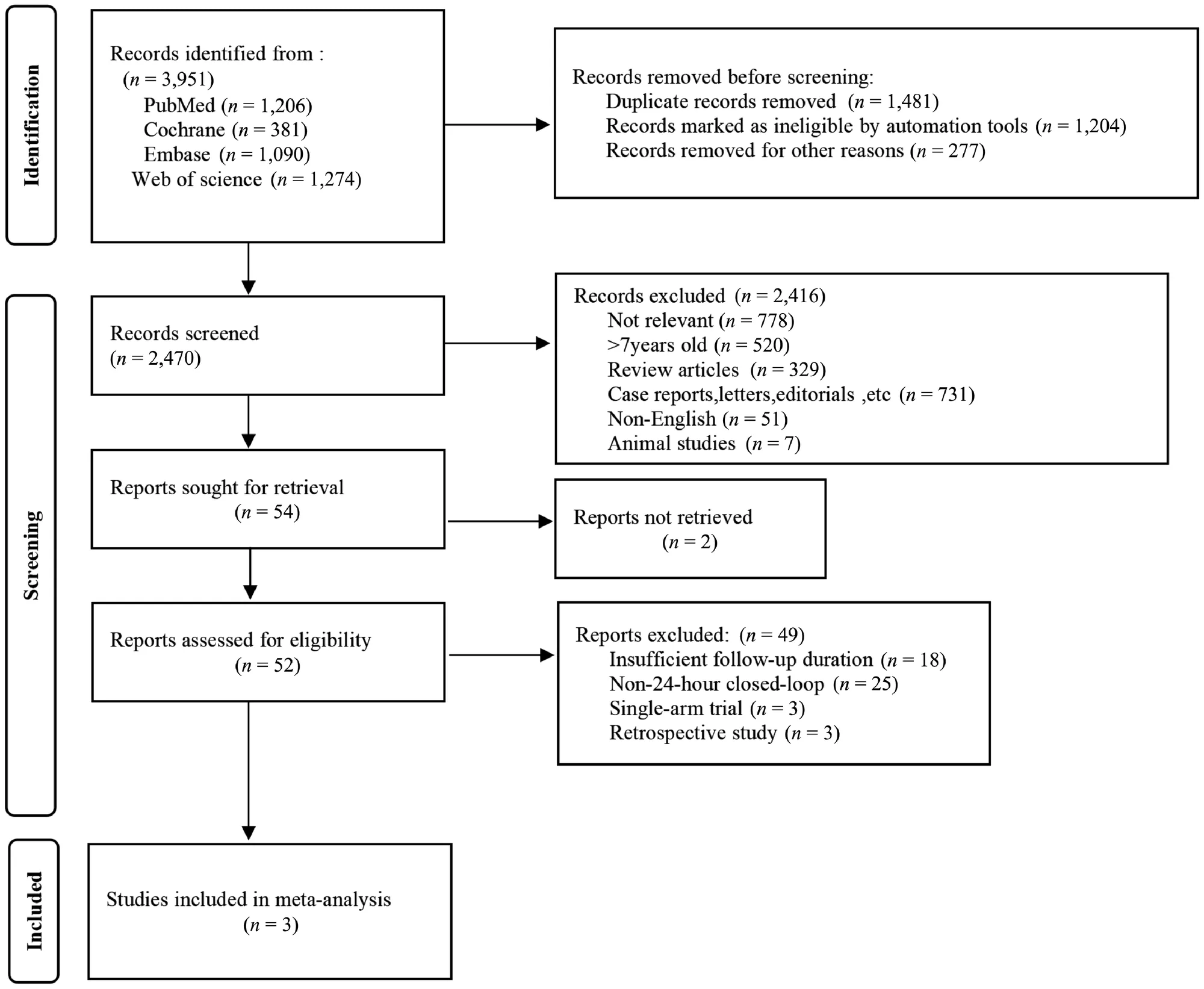
PRISMA flowchart for study selection.

### Study characteristics

Across the three included studies, two were randomized crossover controlled trials and one used a parallel-group controlled design. The intervention groups utilized a HCL system for 24-h glycemic management, whereas the control groups received conventional insulin therapy, including the manual mode of the MiniMed 780G system, the manual mode of the CamAPS FX system, CSII (Continuous Subcutaneous Insulin Infusion), and MDI (Multiple Daily Injections). The follow-up period was approximately 3 months. Information such as the basic study characteristics, the participants' demographics, intervention and control measures, and baseline HbA1c levels were extracted, as detailed in Table 1.

**Table 1:** Basic characteristics of the studies.

Study	Journal	Study design	Number of patients	Age, years (mean ± SD)	Females, *n* (%)	Prior therapy	Type of HCL	Control group treatment	Baseline HbA1c (%) mean ± SD		Study duration
									HCL	Control	
Battelino et al.^[22]^	*Lancet Diabetes Endocrinol*	RCRT	98	4.7 ± 1.2	48 (49%)	MDI: 21%, CSII: 8%, SAP: 70%	MiniMed 780G system	MM + SBL	7.5 ± 1.2	7.7 ± 0.9	Two 12-week periods
Wadwa et al.^[23]^	*N Engl J Med*	RCT	102	3.9 ± 1.2	52 (51%)	CSII: 65%, MDI: 35%	Tandem control-IQ	MDI or CSII + CGM	7.3 ± 0.7	7.4 ± 0.6	13-week
Ware et al.^[24]^	*N Engl J Med*	RCRT	74	5.6 ± 1.6	31 (42%)	CSII	CamAPS FX	MM + SAP	7.50 ± 0.91	7.56 ± 1.01	Two 16-week periods
RCRT, randomized crossover trial; SBL, sensor-based low glucose suspension; AM, auto mode; MM, manual + SBL mode; SAP, sensor-augmented pump.											

### Results of the synthesis

#### Primary outcome (TIR)

Three included studies reported the time TIR. The heterogeneity analysis revealed no statistically significant heterogeneity across the included studies (I^2^ = 28%, χ^2^ = 2.79, df = 2, *p* = 0.25). The random effects model indicated that TIR was significantly higher in the HCL group compared with the controls. The MD was 9.95% (95% CI: 7.80 to 12.09), which was statistically significant (*p* < 0.00001) (Fig. 2a). Sensitivity analysis, performed by sequentially excluding each included study, showed that the pooled effect size remained consistent without substantial change (*Z*-values ranged from 4.59 to 9.09, *p* < 0.00001).

**Figure 2:**
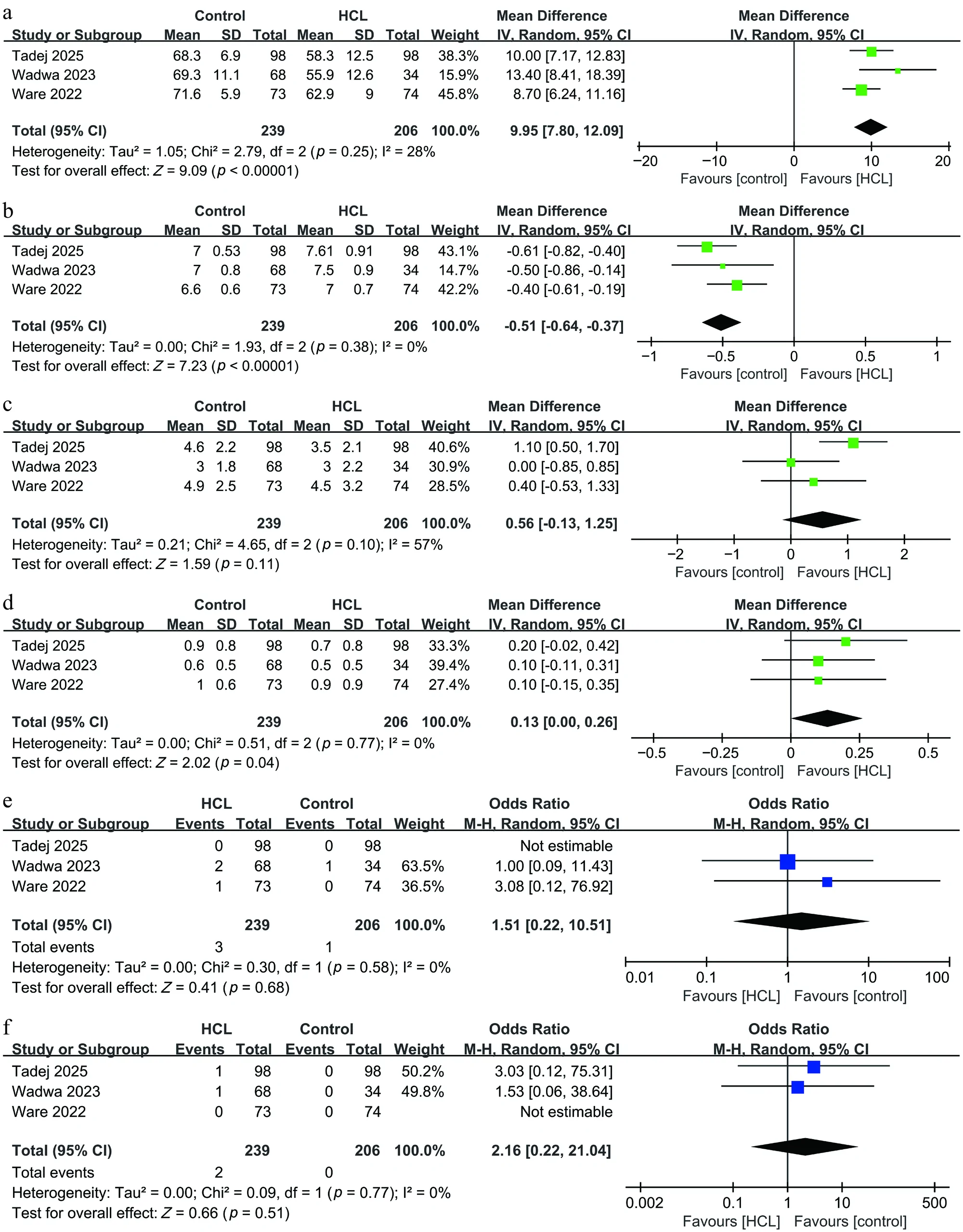
Forest plots of glycemic outcomes and serious adverse events. (a) TIR. (b) HbA1c. (c) Percentage of time with sensor glucose < 70 mg/dL. (d) Percentage of time with sensor glucose < 54 mg/dL. (e) Severe hypoglycemia. (f) DKA.

#### Secondary outcomes

##### HbA1c

All three included studies provided data on HbA1c (%). Heterogeneity testing indicated no heterogeneity across the studies (I^2^ =0%, χ^2^ = 1.93, degrees of freedom [df] = 2, *p* = 0.38). Pooled findings derived showed that the HbA1c was significantly lower in the intervention group than in the conventional insulin therapy group (MD –0.51%, 95% CI [–0.64, –0.37]) (Fig. 2b). The findings were robust across sensitivity analyses, in which the iterative removal of individual studies did not substantially change the estimates of the meta-analysis (*Z*-values ranged from 4.6 to 7.23, *p* < 0.00001).

##### Percentage of time with sensor glucose < 70 mg/dL

Three studies were included in the meta-analysis, each reporting TBR as an outcome measure. The pooled results indicated that the difference in TBR between the HCL and conventional therapy groups was not statistically significant, according to a random-effects model. The MD was 0.56% (95% CI [–0.13, 1.25], *p* = 0.11), as shown in Fig. 2c. However, considerable heterogeneity was detected among the included studies (I^2^ = 57%, χ^2^ = 4.65, df = 2, *p* = 0.1).

Leave-one-out sensitivity analysis was performed, systematically excluding individual studies to assess the robustness of the pooled estimates. After excluding the study by Battelino et al.^[22]^, the difference in TBR between the intervention and conventional insulin therapy groups remained statistically non-significant, with an MD of 0.18% (95% CI [–0.44, 0.81], *p* = 0.57). Heterogeneity remained low, with an I^2^ value of 0% (*p* = 0.53). Upon exclusion of the study by Wadwa et al.^[23]^, heterogeneity among the remaining two studies decreased (I^2 ^= 35%, *p* = 0.21). The pooled effect size became statistically significant, showing an MD of 0.84% (95% CI: 0.18–1.5, *p* = 0.01). When the study by Ware et al. was removed^[24]^, heterogeneity increased substantially to 76%. However, the difference between groups remained nonsignificant, with an MD of 0.59% (95% CI [–0.48, 1.67], *p* = 0.28).

##### Percentage of time with sensor glucose < 54 mg/dL

There was a borderline statistical significant difference between the HCL and conventional insulin therapy, with an MD of 0.13% (95% CI [0.00, 0.26], *p* = 0.04 (Fig. 2d). No significant heterogeneity was observed (I^2^ = 0%, χ^2^ = 0.51, df = 2, *p* = 0.77).

To assess the robustness of the pooled results, sensitivity analysis was conducted by excluding each individual study in sequence. After excluding the study by Battelino et al.^[22]^, the difference in TBR between the intervention and control groups was not statistically significant (MD 0.1%, 95% CI [–0.06, 0.26], *p* = 0.22), with minimal heterogeneity (I^2 ^= 0%). When the study by Wadwa et al. was removed^[23]^, the heterogeneity among the remaining two studies remained unchanged (I^2 ^= 0%, *p* = 0.56), whereas the pooled effect size lost its statistical significance (MD 0.15%, 95% CI [–0.01, 0.32], *p* = 0.07). After excluding the study by Ware et al.^[24]^, the result also became nonsignificant (MD 0.15%, 95% CI [–0.01, 0.30], *p* = 0.06), with substantial heterogeneity detected (I^2^ = 76%).

##### Percentage of time with sensor glucose > 180 mg/dL, >250 mg/dL, and > 300 mg/dL

The percentage of time spent with sensor glucose above the hyperglycemia thresholds was reported in all three included studies, focusing on children aged < 7 years with T1D. comparing those using a HCL system with a control group. The results demonstrated that the HCL group spent a lower percentage of time above all hyperglycemia thresholds compared with the control group, as detailed in Table 2.

**Table 2:** Percentage of time with hyperglycemia.

Study	Sensor glucose	HCL group	Control group	Difference (95%CI)	*p*-value
Battelino et al.^[22]^	> 180 mg/dL	27.1 ± 6.4	38.1 ± 13	NA	NA
	> 250 mg/dL	7.7 ± 4.6	13.1 ± 9.5	NA	NA
Wadwa et al.^[23]^	> 250 mg/dL	8.4 ± 7.2	15.0 ± 10.9	−5.4 (−0.73 to −3.6)	< 0.001
Ware et al.^[24]^	> 180 mg/dL	22.9 (19.3–27.3)	31.7 (23.4–40.1)	−8.5 (−9.9 to −7.1)	< 0.001
	> 300 mg/dL	2 (1.2–3.1)	3.1 (1.3–5.7)	−1.0 (−1.6 to −0.6)	NA
Data are presented as the mean ± standard deviation or median (95% CI); between-group differences, 95% CIs, and *p*-values were obtained from the original studies.					

#### Serious adverse events

The risk of severe hypoglycemia and DKA was pooled using the Mantel-Haenszel (M-H) random-effects model.

##### Severe hypoglycemia

Three studies involving 239 patients in the HCL group and 206 in the control group were included. No significant heterogeneity was detected (I^2^ = 0%, χ^2^ = 0.3, df = 1, *p* = 0.58). The pooled results demonstrated no statistically significant difference in the risk of severe hypoglycemia between the two groups (odds ratio [OR] = 1.51, 95% CI: 0.22–10.51, *p* = 0.68) (Fig. 2e). The effect size for the Ware et al.^[24]^ study was not estimable because there were zero events in both groups.

##### Diabetic ketoacidosis

Data from the same three studies were analyzed. Heterogeneity analysis indicated no between-study heterogeneity (I^2^ = 0%, χ^2^ = 0.09, df = 1, *p* = 0.77). The meta-analysis showed no significant difference in the risk of DKA between the HCL group and the control group (OR = 2.16, 95% CI: 0.22–21.04, *p* = 0.51) (Fig. 2f). The effect of the Battelino et al.'s study^[22]^ was not estimable as no DKA events occurred in either arm.

For both of the abovementioned serious adverse events, a sensitivity analysis was conducted by using the leave-one-out method, whereby the pooled effect size was recalculated after sequentially excluding each individual study. The results demonstrated no substantial changes in the pooled effect estimates or their statistical significance, confirming the robustness of our findings.

### Results of assessing the risk of bias

In relation to the randomization procedure, all included studies outlined suitable approaches for both the generation of random sequences and the concealment of allocation. Because of the inherent nature of the HCL intervention, blinding of the participants and personnel was not feasible, resulting in some worries regarding possible departures from the interventions that were planned or designed. The risk of bias arising from missing outcome data was evaluated as low. Measurements of the outcomes were based on objective sensor or laboratory data, leading to a low risk rating in this domain for all studies. No evidence was found to suggest the presence of selective outcome reporting. Of the three included studies, one was categorized as having a low risk of bias^[23]^, whereas the remaining two were categorized as having a moderate risk of bias^[22,24]^. Details of the bias risk assessment are presented in Supplementary Fig. S1.

### Certainty of the evidence

The certainty of the evidence for key outcomes of HCL therapy versus conventional therapy in children aged < 7 years with T1D was assessed using the GRADE framework. The clinical recommendation strength was determined by synthesizing the certainty of evidence, the benefit–risk balance, and the needs of clinical practice, with the results summarized in Table 3.

**Table 3:** Summary of the findings (GRADE)

Certainty assessment									No. of patients			Effect		Certainty	Importance
Outcome	No. of studies	Study design	Risk of bias	Inconsistency	Indirectness	Imprecision	Other considerations		HCL	Conventional therapy		Relative (95% CI)	Absolute (95% CI)		
Time in range (sensor glucose 70–180 mg/dL, TIR)	3	RCT	Not serious	Not serious	Not serious	Serious^a^	None		239	206		–	MD 9.95 higher (7.80–12.09 higher)	⊕⊕⊕○ Moderate^a^	Critical
Glycated hemoglobin (HbA1c)	3	RCT	Not serious	Not serious	Not serious	Serious^a^	None		239	206		–	MD 0.51 lower (0.64–0.37 lower)	⊕⊕⊕○ Moderate^a^	Critical
Percentage of time with sensor glucose < 70 mg/dL	3	RCT	Not serious	Serious^b^	Not serious	serious^a^	None		239	206		–	MD 0.56 higher (0.13 lower to 1.25 higher)	⊕⊕○○ Low^a,b^	Important
Percentage of time with sensor glucose < 54 mg/dL	3	RCT	Not serious	Not serious	Not serious	Serious^a^	None		239	206		–	MD 0.13 higher (0.00–0.26 higher)	⊕⊕⊕○ Moderate^a^	Important
Diabetic ketoacidosis	3	RCT	Not serious	Not serious	Not serious	Serious^c^	none		239 (2 events)	206 (0 events)		OR 2.16 (0.22–21.04)	0.8% in HCL vs 0.0% in control	⊕⊕⊕○ Moderate^c^	Critical
Severe hypoglycemia	3	RCT	Not serious	Not serious	Not serious	Serious^c^	None		239 (3 events)	206 (1 event)		OR 1.51 (0.22–10.51)	7.6 more per 1,000 (from 4 fewer to 44 more)	⊕⊕⊕○ Moderate^c^	Critical
CI: confidence interval; MD: mean difference; OR: odds ratio; HCL: hybrid closed-loop; TIR: time in range; RCT: randomized controlled trial. a. A small number of included studies (3 RCTs) and limited total sample size. b. I^2^ = 57%. c. Low event rate and wide confidence intervals, causing imprecision.															

#### Glycemic control (TIR and HbA1c)

Certainty of evidence: Moderate (⊕⊕⊕○, downgraded for imprecision because of the limited sample size).

Recommendation strength: Strong recommendation for HCL therapy. HCL therapy significantly increased TIR and reduced HbA1c, with clear and clinically meaningful benefits for glycemic control, which is of great value for long-term glycemic management in children.

#### Percentage of time with sensor glucose < 54 mg/dL

Certainty of evidence: Moderate (⊕⊕⊕○, downgraded for imprecision).

Recommendation strength: Evidence-based practice statement. A slight nonsignificant increase was observed in the HCL group (MD = 0.13%, 95% CI: 0.00–0.26), with unclear clinical significance, requiring continuous monitoring of this indicator.

#### Percentage of time with sensor glucose < 70 mg/dL

Certainty of evidence: Low (⊕⊕○○, downgraded for imprecision and serious inconsistency [I^2^ = 57%]).

Recommendation strength: Evidence-based practice statement. No statistically significant difference was found between the two groups (MD = 0.56%, 95% CI: –0.13 to 1.25), with insufficient evidence for the effect of HCL therapy on this indicator.

#### Adverse events (DKA and severe hypoglycemia)

Certainty of evidence: moderate (⊕⊕⊕○, downgraded for imprecision because of a low event rate).

Recommendation strength: strong recommendation for HCL therapy combined with standardized safety education.

GRADE certainty assessment was not performed for hyperglycemia-related outcomes. Because of the insufficient number of included studies, only a descriptive analysis was conducted, and effective data pooling was not carried out, and thus these outcomes were not included in the GRADE evaluation of the core outcomes.

## Discussion

This meta-analysis systematically synthesized data from three RCTs to evaluate the efficacy of HCL systems on glycemic control and safety outcomes in children under 7 years of age with T1D. The key findings are summarized as follows. For core glycemic control measures, the HCL group had a significantly higher TIR (70–180 mg/dL) and a significantly lower HbA1c level compared with the control group, with stable and reliable pooled results and low between-study heterogeneity for both indicators. For hypoglycemia-related outcomes, no consistent statistically significant differences were found between the two groups in the TBR for sensor glucose < 70 mg/dL and < 54 mg/dL. Notably, significant heterogeneity was identified in the TBR for sensor glucose < 70 mg/dL (I^2 ^= 57%), and sensitivity analyses suggested that individual studies had a certain impact on the pooled results of this indicator. For hyperglycemia-related measures, the HCL group had a lower time above range at the thresholds of > 180 mg/dL, > 250 mg/dL, and > 300 mg/dL than the control group, with statistically significant differences observed for some of these indicators. However, heterogeneity tests and meta-analytic pooling were not conducted for these outcomes because of an insufficient number of included studies (≤ 2). In terms of safety, the incidence of SAEs, including severe hypoglycemia and DKA, was low in both groups, with no significant intergroup differences detected. Collectively, HCL systems effectively improve glycemic control in children under 7 years with T1D without elevating the risk of adverse events. Nevertheless, the results for some hypoglycemia-related indicators lack adequate stability, highlighting the need for further validation with more high-quality clinical studies.

Young children present unique challenges in diabetes management because of their unpredictable eating and activity patterns, higher insulin sensitivity, and greater variability in insulin requirements^[25,26]^. Minimizing the hypoglycemia risk while maintaining tight glycemic control is particularly challenging. Maintaining optimal glucose levels is crucial for brain growth and neurocognitive development during early childhood^[27]^. As the core metric of continuous glucose monitoring (CGM) recommended by international consensus, TIR has been formally incorporated into the clinical practice guidelines of authoritative international organizations such as the American Diabetes Association (ADA) and the International Society for Pediatric and Adolescent Diabetes (ISPAD), which defines its irreplaceable role in the glycemic management of pediatric T1D^[28]^. Unlike HbA1c, which merely reflects the long-term average glycemic level, TIR dynamically quantifies the proportion of time that blood glucose remains within the target range over a specific period, which can more accurately capture the real-time glycemic fluctuation characteristics and avoid the limitation of HbA1c in ignoring transient glycemic extremes. Research by Gaudieri et al. suggests that extreme glycemic fluctuations may have potential adverse effects on neurocognitive function in young children during critical periods of rapid brain development^[29]^. This is especially relevant for children diagnosed before the age of 7, who are more likely to exhibit impairments in cognitive domains such as verbal ability, visual learning, and memory^[30]^. More importantly, accumulating clinical evidence has confirmed that TIR is independently associated with the progression of diabetes-related microvascular complications: each 5% incremental increase in TIR is correlated with significant reductions in the incidence of diabetic retinopathy, nephropathy, and peripheral neuropathy in pediatric T1D, which is a more sensitive predictor of long-term complication risk than HbA1c in this vulnerable population^[28]^.

The results indicated that in children aged < 7 years with T1D, the use of a HCL system for over 3 months resulted in a 9.95% increase in TIR, equivalent to approximately 2.4 additional hours per day spent within the target glycemic range. This 9.95% increment in TIR, approaching the threshold of a 10% increase, has substantial clinical significance for this specific population: it not only reflects the optimized precision of daily glycemic management achieved by HCL systems but also translates to a clinically meaningful reduction in long-term complication risks. This improvement is of major clinical importance for delaying the onset of diabetes-related microvascular complications and mitigating the potential adverse effects of glycemic variability on the central nervous system's development^[31,32]^. According to the international consensus on TIR, each 5% increase in TIR confers significant clinical benefits for patients with T1D, and a 10% increase is associated with a 13.6% greater reduction in the risk of mild cognitive impairment (MCI) and a marked decrease in the progression of microvascular complications^[28]^. For young children with T1D who face a lifelong disease course, this improvement in TIR is conducive to mitigating the cumulative damage of glycemic fluctuations on the target organs (e.g., brain, retina, and kidneys) and optimizing long-term prognosis. Additionally, the extended TIR can reduce the frequency and amplitude of glycemic fluctuations, thereby alleviating oxidative stress and inflammatory responses induced by glycemic extremes, which further contributes to the protection of vascular endothelial function and neurocognitive integrity^[32]^. This finding is consistent with several authoritative studies and meta-analyses conducted in broader age groups of individuals with T1D. For instance, a meta-analysis by Weisman et al. reported that closed-loop systems generally increase TIR by approximately 12.59%. Similarly, another study documented a comparable improvement in TIR of around 9.62%^[33,34]^. A study by Qin Yang et al. indicated that HCL use was associated with an average TIR increase of 11.61% compared with conventional insulin therapy^[35]^. Other studies specifically focused on children and adolescents demonstrated that the use of HCL system increased TIR by 10.89%^[36]^. Although these studies included older children, evidence from various age groups and study designs consistently indicates that HCL technology serves as a powerful tool for improving TIR. The present meta-analysis confirms that even in very young children, HCL remain effective in addressing management challenges and significantly enhancing glycemic time in range.

Second, the results demonstrated that the HCL system significantly reduced HbA1c levels by 0.51%. This reduction in HbA1c, consistent with the observed improvement in TIR, confirms the superiority of HCL in long-term glycemic control. This finding aligns with multiple studies reporting similar reductions of approximately 0.5%^[36−39]^. Weisman et al. reported a slightly lower reduction of around 0.3%^[34]^. Even modest reductions in HbA1c, particularly at relatively low baseline levels, are associated with long-term benefits. Therefore, the 0.51% decrease observed suggests a substantial reduction in the risk of long-term complications in this population. It is particularly noteworthy that achieving a reduction of this magnitude is clinically challenging in populations with relatively low baseline HbA1c levels (as observed in the included studies, where the mean baseline values ranged from 7.3% to 7.7%). This further underscores the advantage of HCL systems in optimizing glycemic control^[40,41]^.

For the analysis of hypoglycemia, two distinct thresholds were defined: the percentage of time when sensor glucose fell to < 70 mg/dL and the percentage when they were at < 54 mg/dL. The meta-analysis showed no significant difference in TBR between the two groups. However, considerable heterogeneity was detected across studies, indicating the limited robustness of this outcome. The statistical significance was altered upon exclusion of the study by Wadwa et al.^[23]^, whereas removal of the study by Ware et al. resulted in a notable increase in heterogeneity^[24]^. The following factors may explain the observed variability: (1) The three included studies utilized three different HCL systems, each using distinct control algorithms. The MiniMed 780G system incorporates a proportional integral derivative (PID) algorithm with insulin feedback, whereas the Tandem Control-IQ and CamAPS FX systems utilize model predictive control (MPC). Differences in the control strategies may contribute to inconsistencies in the efficacy of hypoglycemia prevention. This is particularly relevant in young children, who exhibit high insulin sensitivity and a narrow therapeutic window for insulin dosing^[42]^. Rapid fluctuations in physical activity, emotional state, and growth hormone secretion further complicate accurate prediction of insulin requirements, necessitating highly adaptable algorithms capable of maintaining glucose within a tight target range^[43]^. (2) Variations in target glucose levels and low-glucose thresholds for suspension across HCL systems may have influenced the outcomes^[44]^. (3) Heterogeneity in trial design was observed: two studies adopted a randomized crossover design, whereas the third used a parallel-group design, introducing inherent methodological diversity. (4) Insulin therapy regimens in the control groups were not uniform across studies. (5) The small quantity of included studies, coupled with the insufficient overall sample size, undermined the robustness of the pooled estimate.

Regarding the percentage of time spent wth sensor glucose < 54 mg/dL, the initial pooled analysis indicated a statistically significant discrepancy between the two groups. However, the lower bound of the confidence interval was 0.00% (*p* = 0.04), approaching the threshold for nonsignificance. More importantly, sensitivity analysis revealed that the removal of any single study resulted in a loss of statistical significance, diminishing the robustness of this marginally significant finding. This suggests that the HCL system may neither significantly increase nor decrease the incidence of severe hypoglycemia. This observation is consistent with the majority of studies conducted in adults and older children^[16,34,45−47]^. Through its predictive low glucose suspension feature, the HCL system can effectively prevent prolonged severe hypoglycemia by mitigating a sustained decline in glucose; however, it may not completely eliminate transient hypoglycemic events that have already fallen below the threshold^[45]^. In conclusion, HCL therapy did not significantly increase the risk of severe hypoglycemia in children under 7 years old.

In addition to hypoglycemia risk, the level of glycemic control in hyperglycemia serves as another key indicator for evaluating the effectiveness of HCL. Because of inconsistencies in the hyperglycemia thresholds used across the three included RCTs, a pooled quantitative analysis was not performed. In the study by Battelino et al.^[22]^, the percentage of time with sensor glucose > 180 mg/dL was significantly lower in the HCL group (27.1%) than in the open-closed group (38.1%), a result consistent with the findings reported by Ware et al.^[24]^. Furthermore, the HCL system demonstrated advantages in managing severe hyperglycemia. At the threshold of > 250 mg/dL, the study by Wadwa et al. reported an absolute reduction of 5.4%^[23]^, and a consistent trend was observed in the study by Battelino et al. (HCL: 7.7% vs control: 13.1%)^[22]^. At an even higher threshold of > 300 mg/dL, the study by Ware et al. showed a lower time percentage in the HCL group (2% vs 3.1%)^[24]^. For children under 7 years of age, who often have limited ability to express symptoms and are more susceptible to dehydration, preventing DKA is critically important. The HCL system provides automated correction doses and dynamically adjusts basal insulin delivery, enabling more timely and effective responses to hyperglycemia. This offers an additional layer of safety for this vulnerable population and helps alleviate anxiety and stress among caregivers^[14]^. By optimizing insulin delivery, the HCL system redistributes glucose levels from the hyperglycemic range to the target range, thereby achieving improved glycemic control^[31]^.

Although glycemic improvement is important, safety remains a critical consideration. SAEs serve as a key metric for safety evaluation. Data indicated that severe hypoglycemic events occurred sporadically in both the HCL and control groups, with a very low overall incidence (four events in total) and no significant difference between the groups. This suggests that HCL systems do not increase the risk of severe hypoglycemia. Although the total number of adverse events was numerically higher in the HCL group, the difference did not reach statistical significance. This indicates that the HCL system did not significantly increase the overall risk of adverse events compared with conventional therapy, which is consistent with the findings reported by Tauschmann et al.^[42]^. Across the three studies, two DKA events were reported in the HCL groups, one of which was related to an infusion set failure. Notably, this risk of DKA caused by the infusion system's failure also exists in CSII. In fact, by virtue of its continuous glucose monitoring and early alert functions, the HCL system may enable earlier detection of a rapid glucose elevation compared with traditional pump therapy, thereby allowing more timely intervention. No DKA occurrences were recorded in the control group; however, given the limited sample size, this should not be interpreted as indicating a lower risk in the control group.

This meta-analysis provides the first pooled evidence demonstrating that HCL systems can significantly improve glycemic control in children aged < 7 years, thereby offering evidence-based support for their clinical use in this vulnerable population. The analysis encompassed key glycemic metrics (TIR and HbA1c) as well as safety outcomes (TBR, TAR, and adverse events), allowing a comprehensive and multidimensional evaluation of the efficacy and safety of HCL systems.

The study is not without limitations. The small number of included RCTs and the limited sample size resulted in reduced statistical power, unstable estimates of I^2^, and limited ability to assess publication bias. The study duration was approximately 3 months, which precludes assessment of long-term safety outcomes. Restricting inclusion to English publications may have omitted relevant studies published in other languages. Additionally, because of insufficient data, no subgroup analysis of daytime versus nighttime glycemic control was performed^[42,48]^.

Future research should prioritize large-sample, multicenter randomized controlled trials with prolonged follow-up durations to validate these findings and provide long-term safety and efficacy data. There is also a need for real-world studies to evaluate the applicability and effectiveness of HCL systems across diverse healthcare settings and socioeconomic backgrounds.

## Conclusions

In conclusion, HCL therapy significantly improves TIR and HbA1c control in children aged < 7 years with TID compared with control therapies, without increasing the risk of hypoglycemia and adverse events. These findings provide support for the efficacy and safety of HCL systems in treating T1D. However, further long-term RCTs are warranted to upport these findings.

## SUPPLEMENTARY DATA

Supplementary data to this article can be found online.

## Data Availability

The data that support the findings of this study are available on request from the corresponding author.
