# Supplementary material for: Efficacy and safety of hybrid closed-loop systems in children aged < 7 years with type 1 diabetes: a systematic review and meta-analysis of randomized controlled trials
Source: Pediatr Diabetes. 2026 Aug 12;2026:e006. doi: 10.48130/pedi-0026-0006 (PMC13599426; doi:10.48130/pedi-0026-0006)
Supplement: Supplementary file 1 [file pedi-2026-2026-1-0006-S1.zip › 10.48130_pedi-0026-0006-Suppl-File1.docx]

**Supplementary File S1：Search strategies.**

**PubMed**

# ****MeSH Terms :**** Pancreas, Artificial

### ****Keywords:****Artificial Pancreas, Automated insulin delivery system, Bionic Pancreas, Closed-Loop Control, Closed-Loop Insulin, Hybrid Closed-Loop Control, Closed Loop Insulin Delivery

### ****MeSH Terms :**** Child

**Keyword**: Children

****MeSH Terms :**** Diabetes Mellitus, Type 1

****Keywords:**** Type 1 Diabetes, Diabetes, Type 1, Diabetes Mellitus, Insulin-Dependent, Diabetes Mellitus, Insulin Dependent, Insulin-Dependent Diabetes Mellitus, Diabetes Mellitus, Juvenile-Onset, Diabetes Mellitus, Juvenile Onset, Juvenile-Onset Diabetes Mellitus, IDDM, Diabetes Mellitus, Type I, Diabetes Mellitus, Sudden-Onset, Diabetes Mellitus, Sudden Onset, Sudden-Onset Diabetes Mellitus, Type 1 Diabetes Mellitus, Diabetes Mellitus, Insulin-Dependent, 1 , Insulin-Dependent Diabetes Mellitus 1, Insulin Dependent Diabetes Mellitus 1, Juvenile-Onset Diabetes, Diabetes, Juvenile-Onset, Juvenile Onset Diabetes, Diabetes, Autoimmune ,Autoimmune Diabetes, Diabetes Mellitus, Brittle, Brittle Diabetes Mellitus, Diabetes Mellitus, Ketosis-Prone, Diabetes Mellitus, Ketosis Prone, Ketosis-Prone Diabetes Mellitus

**Complete Search Syntax**

("pancreas, artificial"[MeSH Terms] OR ("pancreas, artificial"[MeSH Terms] OR ("pancreas"[All Fields] AND "artificial"[All Fields]) OR "artificial pancreas"[All Fields] OR ("artificial"[All Fields] AND "pancreas"[All Fields]) OR ("appl phys kowloon"[Journal] OR "anal psychol"[Journal] OR "alpha psychiatry"[Journal] OR "actual psicol"[Journal] OR "ap"[All Fields]) OR ("pancreas, artificial"[MeSH Terms] OR ("pancreas"[All Fields] AND "artificial"[All Fields]) OR "artificial pancreas"[All Fields] OR ("automated"[All Fields] AND "insulin"[All Fields] AND "delivery"[All Fields] AND "system"[All Fields]) OR "automated insulin delivery system"[All Fields]) OR "AID"[All Fields] OR (("bionics"[MeSH Terms] OR "bionics"[All Fields] OR "bionic"[All Fields]) AND ("pancrea"[All Fields] OR "pancreas"[MeSH Terms] OR "pancreas"[All Fields])) OR ("Closed-Loop"[All Fields] AND ("controling"[All Fields] OR "controllability"[All Fields] OR "controllable"[All Fields] OR "controllably"[All Fields] OR "controller"[All Fields] OR "controller s"[All Fields] OR "controllers"[All Fields] OR "controlling"[All Fields] OR "controls"[All Fields] OR "prevention and control"[MeSH Subheading] OR ("prevention"[All Fields] AND "control"[All Fields]) OR "prevention and control"[All Fields] OR "control"[All Fields] OR "control groups"[MeSH Terms] OR ("control"[All Fields] AND "groups"[All Fields]) OR "control groups"[All Fields])) OR ("Closed-Loop"[All Fields] AND ("insulin"[Supplementary Concept] OR "insulin"[All Fields] OR "insulin"[MeSH Terms] OR "insulin s"[All Fields] OR "insuline"[All Fields] OR "insulinic"[All Fields] OR "insulinization"[All Fields] OR "insulinized"[All Fields] OR "insulins"[MeSH Terms] OR "insulins"[All Fields])) OR (("chimera"[MeSH Terms] OR "chimera"[All Fields] OR "hybrid"[All Fields] OR "hybrids"[All Fields] OR "hybrid s"[All Fields] OR "hybridation"[All Fields] OR "hybridisations"[All Fields] OR "hybridise"[All Fields] OR "hybridised"[All Fields] OR "hybridises"[All Fields] OR "hybridising"[All Fields] OR "hybridity"[All Fields] OR "hybridization, genetic"[MeSH Terms] OR ("hybridization"[All Fields] AND "genetic"[All Fields]) OR "genetic hybridization"[All Fields] OR "hybridisation"[All Fields] OR "hybridizations"[All Fields] OR "hybridize"[All Fields] OR "hybridized"[All Fields] OR "hybridizes"[All Fields] OR "hybridizing"[All Fields] OR "nucleic acid hybridization"[MeSH Terms] OR ("nucleic"[All Fields] AND "acid"[All Fields] AND "hybridization"[All Fields]) OR "nucleic acid hybridization"[All Fields] OR "hybridization"[All Fields]) AND "Closed-Loop"[All Fields] AND ("controling"[All Fields] OR "controllability"[All Fields] OR "controllable"[All Fields] OR "controllably"[All Fields] OR "controller"[All Fields] OR "controller s"[All Fields] OR "controllers"[All Fields] OR "controlling"[All Fields] OR "controls"[All Fields] OR "prevention and control"[MeSH Subheading] OR ("prevention"[All Fields] AND "control"[All Fields]) OR "prevention and control"[All Fields] OR "control"[All Fields] OR "control groups"[MeSH Terms] OR ("control"[All Fields] AND "groups"[All Fields]) OR "control groups"[All Fields])) OR (("close"[All Fields] OR "closed"[All Fields] OR "closely"[All Fields] OR "closeness"[All Fields] OR "closes"[All Fields] OR "closing"[All Fields] OR "closings"[All Fields]) AND "Loop"[All Fields] AND ("insulin"[Supplementary Concept] OR "insulin"[All Fields] OR "insulin"[MeSH Terms] OR "insulin s"[All Fields] OR "insuline"[All Fields] OR "insulinic"[All Fields] OR "insulinization"[All Fields] OR "insulinized"[All Fields] OR "insulins"[MeSH Terms] OR "insulins"[All Fields]) AND ("deliveries"[All Fields] OR "delivery, obstetric"[MeSH Terms] OR ("delivery"[All Fields] AND "obstetric"[All Fields]) OR "obstetric delivery"[All Fields] OR "delivery"[All Fields])) OR ("MiniMed"[All Fields] AND "670G"[All Fields]))) AND ("diabetes mellitus, type 1"[MeSH Terms] OR "type 1 diabetes mellitus"[All Fields] OR "type 1 diabetes"[All Fields] OR ("diabetes mellitus, type 1"[MeSH Terms] OR "type 1 diabetes mellitus"[All Fields] OR "diabetes type 1"[All Fields]) OR ("diabetes mellitus, type 1"[MeSH Terms] OR "type 1 diabetes mellitus"[All Fields] OR ("diabetes"[All Fields] AND "mellitus"[All Fields] AND "insulin"[All Fields] AND "dependent"[All Fields]) OR "diabetes mellitus insulin dependent"[All Fields]) OR ("diabetes mellitus, type 1"[MeSH Terms] OR "type 1 diabetes mellitus"[All Fields] OR ("diabetes"[All Fields] AND "mellitus"[All Fields] AND "insulin"[All Fields] AND "dependent"[All Fields]) OR "diabetes mellitus insulin dependent"[All Fields]) OR ("diabetes mellitus, type 1"[MeSH Terms] OR "type 1 diabetes mellitus"[All Fields] OR ("insulin"[All Fields] AND "dependent"[All Fields] AND "diabetes"[All Fields] AND "mellitus"[All Fields]) OR "insulin dependent diabetes mellitus"[All Fields]) OR ("diabetes mellitus, type 1"[MeSH Terms] OR "type 1 diabetes mellitus"[All Fields] OR ("diabetes"[All Fields] AND "mellitus"[All Fields] AND "juvenile"[All Fields] AND "onset"[All Fields]) OR "diabetes mellitus juvenile onset"[All Fields]) OR ("diabetes mellitus, type 1"[MeSH Terms] OR "type 1 diabetes mellitus"[All Fields] OR ("diabetes"[All Fields] AND "mellitus"[All Fields] AND "juvenile"[All Fields] AND "onset"[All Fields]) OR "diabetes mellitus juvenile onset"[All Fields]) OR ("diabetes mellitus, type 1"[MeSH Terms] OR "type 1 diabetes mellitus"[All Fields] OR ("juvenile"[All Fields] AND "onset"[All Fields] AND "diabetes"[All Fields] AND "mellitus"[All Fields]) OR "juvenile onset diabetes mellitus"[All Fields]) OR ("diabetes mellitus, type 1"[MeSH Terms] OR "type 1 diabetes mellitus"[All Fields] OR "iddm"[All Fields]) OR ("diabetes mellitus, type 1"[MeSH Terms] OR "type 1 diabetes mellitus"[All Fields] OR "diabetes mellitus type i"[All Fields]) OR ("diabetes mellitus, type 1"[MeSH Terms] OR "type 1 diabetes mellitus"[All Fields] OR ("diabetes"[All Fields] AND "mellitus"[All Fields] AND "sudden"[All Fields] AND "onset"[All Fields])) OR ("diabetes mellitus, type 1"[MeSH Terms] OR "type 1 diabetes mellitus"[All Fields] OR ("diabetes"[All Fields] AND "mellitus"[All Fields] AND "sudden"[All Fields] AND "onset"[All Fields])) OR ("diabetes mellitus, type 1"[MeSH Terms] OR "type 1 diabetes mellitus"[All Fields] OR ("sudden"[All Fields] AND "onset"[All Fields] AND "diabetes"[All Fields] AND "mellitus"[All Fields]) OR "sudden onset diabetes mellitus"[All Fields]) OR ("diabetes mellitus, type 1"[MeSH Terms] OR "type 1 diabetes mellitus"[All Fields]) OR ("diabetes mellitus, type 1"[MeSH Terms] OR "type 1 diabetes mellitus"[All Fields]) OR ("diabetes mellitus, type 1"[MeSH Terms] OR "type 1 diabetes mellitus"[All Fields] OR "insulin dependent diabetes mellitus 1"[All Fields]) OR ("diabetes mellitus, type 1"[MeSH Terms] OR "type 1 diabetes mellitus"[All Fields] OR "insulin dependent diabetes mellitus 1"[All Fields]) OR ("diabetes mellitus, type 1"[MeSH Terms] OR "type 1 diabetes mellitus"[All Fields] OR ("juvenile"[All Fields] AND "onset"[All Fields] AND "diabetes"[All Fields]) OR "juvenile onset diabetes"[All Fields]) OR ("diabetes mellitus, type 1"[MeSH Terms] OR "type 1 diabetes mellitus"[All Fields] OR ("diabetes"[All Fields] AND "juvenile"[All Fields] AND "onset"[All Fields]) OR "diabetes juvenile onset"[All Fields]) OR ("diabetes mellitus, type 1"[MeSH Terms] OR "type 1 diabetes mellitus"[All Fields] OR ("juvenile"[All Fields] AND "onset"[All Fields] AND "diabetes"[All Fields]) OR "juvenile onset diabetes"[All Fields]) OR ("diabetes mellitus, type 1"[MeSH Terms] OR "type 1 diabetes mellitus"[All Fields] OR ("diabetes"[All Fields] AND "autoimmune"[All Fields]) OR "diabetes autoimmune"[All Fields]) OR ("diabetes mellitus, type 1"[MeSH Terms] OR "type 1 diabetes mellitus"[All Fields] OR ("autoimmune"[All Fields] AND "diabetes"[All Fields]) OR "autoimmune diabetes"[All Fields]) OR ("diabetes mellitus, type 1"[MeSH Terms] OR "type 1 diabetes mellitus"[All Fields] OR ("diabetes"[All Fields] AND "mellitus"[All Fields] AND "brittle"[All Fields]) OR "diabetes mellitus brittle"[All Fields]) OR ("diabetes mellitus, type 1"[MeSH Terms] OR "type 1 diabetes mellitus"[All Fields] OR ("brittle"[All Fields] AND "diabetes"[All Fields] AND "mellitus"[All Fields]) OR "brittle diabetes mellitus"[All Fields]) OR ("diabetes mellitus, type 1"[MeSH Terms] OR "type 1 diabetes mellitus"[All Fields] OR ("diabetes"[All Fields] AND "mellitus"[All Fields] AND "ketosis"[All Fields] AND "prone"[All Fields])) OR ("diabetes mellitus, type 1"[MeSH Terms] OR "type 1 diabetes mellitus"[All Fields] OR ("diabetes"[All Fields] AND "mellitus"[All Fields] AND "ketosis"[All Fields] AND "prone"[All Fields])) OR ("diabetes mellitus, type 1"[MeSH Terms] OR "type 1 diabetes mellitus"[All Fields] OR ("ketosis"[All Fields] AND "prone"[All Fields] AND "diabetes"[All Fields] AND "mellitus"[All Fields]) OR "ketosis prone diabetes mellitus"[All Fields]) OR "diabetes mellitus, type 1"[MeSH Terms]) AND ("Child"[MeSH Terms] OR ("Child"[MeSH Terms] OR "Child"[All Fields] OR "children"[All Fields] OR "child s"[All Fields] OR "children s"[All Fields] OR "childrens"[All Fields] OR "childs"[All Fields]))

**Cochrane**

**Pancreas, Artificial**

Artificial Pancreas

Automated insulin delivery system

Bionic Pancreas

Closed-Loop Control

Closed-Loop Insulin

Hybrid Closed-Loop Control

Closed Loop Insulin Delivery

# **Child**

Children

**Diabetes Mellitus, Type 1**

Type 1 Diabetes

Diabetes, Type 1

Diabetes Mellitus, Insulin-Dependent

Diabetes Mellitus, Insulin Dependent

Insulin-Dependent Diabetes Mellitus

Diabetes Mellitus, Juvenile-Onset

Diabetes Mellitus, Juvenile Onset

Juvenile-Onset Diabetes Mellitus

IDDM

Diabetes Mellitus, Type I

Diabetes Mellitus, Sudden-Onset

Diabetes Mellitus, Sudden Onset

Sudden-Onset Diabetes Mellitus

Type 1 Diabetes Mellitus

Diabetes Mellitus, Insulin-Dependent, 1

Insulin-Dependent Diabetes Mellitus 1

Insulin Dependent Diabetes Mellitus 1

Juvenile-Onset Diabetes

Diabetes, Juvenile-Onset

Juvenile Onset Diabetes

Diabetes, Autoimmune

Autoimmune Diabetes

Diabetes Mellitus, Brittle

Brittle Diabetes Mellitus

Diabetes Mellitus, Ketosis-Prone

Diabetes Mellitus, Ketosis Prone

Ketosis-Prone Diabetes Mellitus

**EMBASE**

**closed loop insulin delivery**

'closed loop insulin pump therapy' OR 'closed loop insulin pump treatment' OR 'closed loop insulin therapy' OR 'closed loop insulin treatment' OR 'closed loop insulin delivery'

**artificial pancreas**

'artificial endocrine pancreas' OR 'artificial pancreas islet' OR 'endocrine pancreas, artificial' OR 'pancreas, artificial' OR 'pancreas, artificial endocrine' OR 'artificial pancreas'

**Child***

**insulin dependent diabetes mellitus**

'diabetes mellitus type 1' OR 'diabetes mellitus type I' OR 'diabetes mellitus, insulin dependent' OR 'diabetes mellitus, insulin-dependent' OR 'diabetes mellitus, juvenile onset' OR 'diabetes mellitus, type 1' OR 'diabetes mellitus, type I' OR 'diabetes type 1' OR 'diabetes type I' OR 'diabetes, juvenile' OR 'DM 1' OR 'early onset diabetes mellitus' OR 'IDDM' OR 'IDDM1' OR 'insulin dependent diabetes' OR 'insulin-dependent diabetes mellitus' OR 'juvenile diabetes' OR 'juvenile diabetes mellitus' OR 'juvenile onset diabetes' OR 'juvenile onset diabetes mellitus' OR 'mckusick 22210' OR 'T1DM' OR 'type 1 diabetes' OR 'type 1 diabetes mellitus' OR 'type I diabetes' OR 'type I diabetes mellitus' OR 'insulin dependent diabetes mellitus'

**Complete Search Syntax**

('closed loop insulin pump therapy'/exp OR 'closed loop insulin pump therapy' OR 'closed loop insulin pump treatment'/exp OR 'closed loop insulin pump treatment' OR 'closed loop insulin therapy'/exp OR 'closed loop insulin therapy' OR 'closed loop insulin treatment'/exp OR 'closed loop insulin treatment' OR 'closed loop insulin delivery'/exp OR 'closed loop insulin delivery' OR 'artificial endocrine pancreas'/exp OR 'artificial endocrine pancreas' OR 'artificial pancreas islet'/exp OR 'artificial pancreas islet' OR 'endocrine pancreas, artificial'/exp OR 'endocrine pancreas, artificial' OR 'pancreas, artificial'/exp OR 'pancreas, artificial' OR 'pancreas, artificial endocrine'/exp OR 'pancreas, artificial endocrine' OR 'artificial pancreas'/exp OR 'artificial pancreas') AND 'child*' AND ('diabetes mellitus type 1'/exp OR 'diabetes mellitus type 1' OR 'diabetes mellitus type i'/exp OR 'diabetes mellitus type i' OR 'diabetes mellitus, insulin dependent'/exp OR 'diabetes mellitus, insulin dependent' OR 'diabetes mellitus, insulin-dependent'/exp OR 'diabetes mellitus, insulin-dependent' OR 'diabetes mellitus, juvenile onset'/exp OR 'diabetes mellitus, juvenile onset' OR 'diabetes mellitus, type 1'/exp OR 'diabetes mellitus, type 1' OR 'diabetes mellitus, type i'/exp OR 'diabetes mellitus, type i' OR 'diabetes type 1'/exp OR 'diabetes type 1' OR 'diabetes type i'/exp OR 'diabetes type i' OR 'diabetes, juvenile'/exp OR 'diabetes, juvenile' OR 'dm 1'/exp OR 'dm 1' OR 'early onset diabetes mellitus'/exp OR 'early onset diabetes mellitus' OR 'iddm'/exp OR 'iddm' OR 'iddm1'/exp OR 'iddm1' OR 'insulin dependent diabetes'/exp OR 'insulin dependent diabetes' OR 'insulin-dependent diabetes mellitus'/exp OR 'insulin-dependent diabetes mellitus' OR 'juvenile diabetes'/exp OR 'juvenile diabetes' OR 'juvenile diabetes mellitus'/exp OR 'juvenile diabetes mellitus' OR 'juvenile onset diabetes'/exp OR 'juvenile onset diabetes' OR 'juvenile onset diabetes mellitus'/exp OR 'juvenile onset diabetes mellitus' OR 'mckusick 22210'/exp OR 'mckusick 22210' OR 't1dm'/exp OR 't1dm' OR 'type 1 diabetes'/exp OR 'type 1 diabetes' OR 'type 1 diabetes mellitus'/exp OR 'type 1 diabetes mellitus' OR 'type i diabetes'/exp OR 'type i diabetes' OR 'type i diabetes mellitus'/exp OR 'type i diabetes mellitus' OR 'insulin dependent diabetes mellitus'/exp OR 'insulin dependent diabetes mellitus')

**Web of science**

(“Pancreas, Artificial” OR "Artificial Pancreas" OR " Automated insulin delivery system" OR " Bionic Pancreas" OR " Closed-Loop Control " OR "Closed-Loop Insulin " OR "Hybrid Closed-Loop Control" OR " Closed Loop Insulin Delivery") AND “Child*” AND (“Diabetes Mellitus, Type 1” OR “Type 1 Diabetes” OR “Diabetes, Type 1” OR “Diabetes Mellitus, Insulin-Dependent” OR “Diabetes Mellitus, Insulin Dependent” OR “Insulin-Dependent Diabetes Mellitus” OR “Diabetes Mellitus, Juvenile-Onset” OR “Diabetes Mellitus, Juvenile Onset” OR “Juvenile-Onset Diabetes Mellitus” OR “IDDM” OR “Diabetes Mellitus, Type I” OR “Diabetes Mellitus,Sudden-Onset” OR “Diabetes Mellitus, Sudden Onset” OR “Sudden-Onset Diabetes Mellitus” OR “Type 1 Diabetes Mellitus” OR “Diabetes Mellitus, Insulin-Dependent, 1” OR “Insulin-Dependent Diabetes Mellitus 1” OR “Insulin Dependent Diabetes Mellitus 1” OR “Juvenile-Onset Diabetes” OR “Diabetes, Juvenile-Onset” OR “Juvenile Onset Diabetes” OR “Diabetes, Autoimmune” OR “Autoimmune Diabetes” OR “Diabetes Mellitus, Brittle” OR “Brittle Diabetes Mellitus” OR “Diabetes Mellitus, Ketosis-Prone” OR “Diabetes Mellitus, Ketosis Prone” OR “Ketosis-Prone Diabetes Mellitus”)
