# Supplementary material for: Efficacy and safety of hybrid closed-loop systems in children aged < 7 years with type 1 diabetes: a systematic review and meta-analysis of randomized controlled trials
Source: Pediatr Diabetes. 2026 Aug 12;2026:e006. doi: 10.48130/pedi-0026-0006 (PMC13599426; doi:10.48130/pedi-0026-0006)
Supplement: Supplementary file 1 [file pedi-2026-2026-1-0006-S1.zip › 10.48130_pedi-0026-0006-Suppl-FigureS1.pdf]

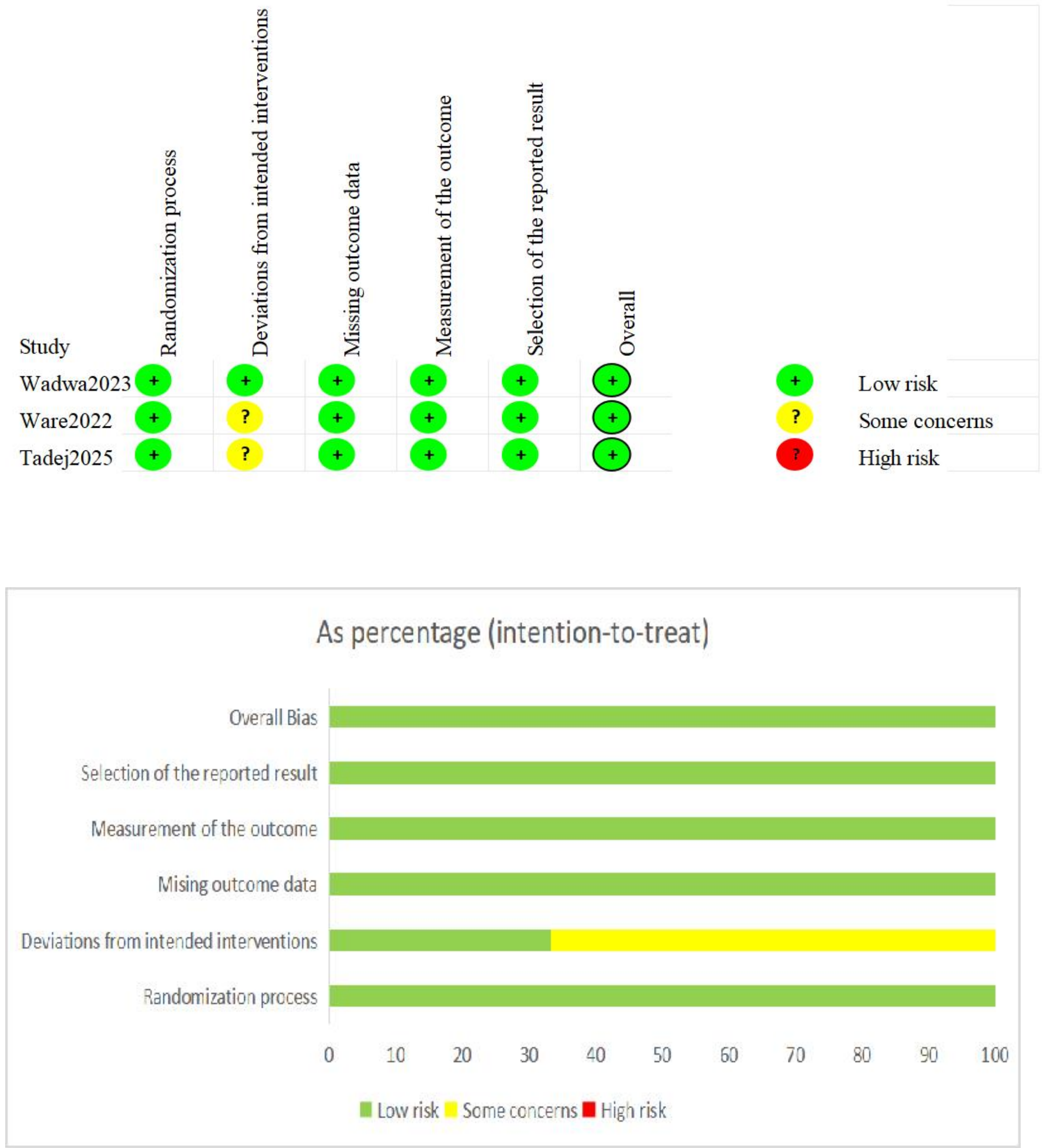

Supplementary Fig. S1: The risks of bias in individual studies and the overall risk of bias.
